# Awareness, uptake, and determinants of germline genetic testing in Chinese epithelial ovarian cancer patients: a cross-sectional study

**DOI:** 10.3389/fonc.2026.1876816

**Published:** 2026-06-19

**Authors:** Xia Wang, Hui-Yuan Cai, Le-Han Lin, Jing You, Yue-Jiao Zhao, Xiao-Min Chen, Hui Wang, Yan Ding

**Affiliations:** 1 Department of Gynecologic Oncology, Obstetrics and Gynecology Hospital of Fudan University, Shanghai Key Lab of Reproduction and Development, Shanghai Key Lab of Female Reproductive Endocrine Related Diseases, Shanghai, China; 2 School of Nursing Fudan University, Shanghai, China; 3 Department of Clinical Research Center, Obstetrics and Gynecology Hospital of Fudan University, Shanghai Key Lab of Reproduction and Development, Shanghai Key Lab of Female Reproductive Endocrine Related Diseases, Shanghai, China

**Keywords:** epithelial ovarian cancer, germline genetic testing, influencing factors, pathogenic variants, testing utilization

## Abstract

**Objective:**

Various guidelines recommend offering germline genetic testing to all patients diagnosed with epithelial ovarian cancer, as it helps guide treatment decisions, identify cancer risks in other organs, and empowers patients to notify families for timely screening and risk-reducing surgery. Our study aimed to explore the awareness and uptake rate of germline genetic testing among Chinese women with epithelial ovarian cancer, and to analyze the determinants influencing their decision.

**Methods:**

A cross sectional study was conducted at Obstetrics and Gynecology Hospital of Fudan University between November 2023 to September 2024. A questionnaire was used to assess patients’ awareness of germline genetic testing. Disease-related data were extracted from electronic medical records. Binary logistic regression analysis was performed to identify factors associated with uptake of germline genetic testing.

**Results:**

Of the total 154 patients included, 100 underwent germline genetic testing, with an uptake rate of 64.94%. Among included patients, 62.99% demonstrated low awareness of germline genetic testing and 37.01% exhibited a high level of awareness. Logistic regression analysis showed that higher monthly per-capita household income (OR = 3.30, 95 % CI 1.24-8.75, P = 0.017), a family history of *BRCA*-related cancers (OR = 6.30, 95% CI 1.16-34.12, P = 0.033), and high awareness of germline genetic testing (OR = 8.81, 95 % CI 2.58-30.12, P = 0.001) were associated with a significantly increased likelihood of undergoing testing. Additionally, 19.6% of the tested patients carried a pathogenic variant.

**Conclusion:**

Among Chinese patients with epithelial ovarian cancer, awareness of germline genetic testing is generally low, with critical deficiencies in knowledge that directly inform clinical decision-making and familial risk management. Nearly two-thirds of patients diagnosed with epithelial ovarian cancer had undergone germline genetic testing. Socioeconomic status, family history of associated cancer, and patients’ awareness of genetic testing are the principal determinants of germline genetic testing utilization in this population.

## Highlights

- Genetic factors are critical in epithelial ovarian cancer, with guidelines recommending universal germline testing for patients. Yet global testing rates remain far below the target due to barriers like cost and limited access. Notably, real-world data on Chinese patients’ testing uptake and determinants remains scarce.
- This study adds that Chinese epithelial ovarian cancer patients have generally low and imbalanced awareness of germline genetic testing, with nearly two-thirds having undergone testing—higher income, *BRCA*-related family cancer history, and greater awareness boost testing likelihood. It also adds that 19.6% of these patients carry pathogenic variants requiring care adjustments.
- This study indicates that targeted strategies are needed to address gaps in timely germline testing for ovarian cancer, including expanding insurance coverage for testing, integrating genetic services into routine care, and notably raising stakeholders’ awareness.

## Introduction

Ovarian cancer remains the most lethal gynecological cancer. It is classified into different histological types, with epithelial ovarian cancer being the most common and having the highest mortality rate. Studies have shown that 15-25% of ovarian cancers are caused by inherited genetic mutations (1–4). A growing body of evidence highlights the critical role of genetic factors in ovarian cancer pathogenesis, particularly pathogenic variants in ovarian cancer susceptibility genes such as *BRCA1*, *BRCA2*, *MLH1*, *MSH2*, *MSH6*, *PMS2*, *EPCAM*, *RAD51C*, *RAD51D*, *BRIP1*, *ATM* and *PALB2* (5). These pathogenic variants not only confer a significantly increased risk of developing ovarian cancer but also influence disease progression, treatment response, and prognosis.

Recognizing the clinical value of genetic information, various clinical guidelines recommend that all patients diagnosed with epithelial ovarian cancer be offered germline genetic testing for ovarian cancer susceptibility genes, irrespective of their clinical features or family cancer history (6–8). Beyond guiding treatment selection, identifying pathogenic variants provides critical insights for holistic patient management: it enables stratification of cancer recurrence risk, screening for second primary malignancies, and, importantly, empowers patients to inform family members. This, in turn, facilitates early familial risk screening and proactive risk-reduction surgery, which are proven to mitigate hereditary cancer burden (9).

Despite these evidence-based recommendations, reported rates of germline genetic testing for people with ovarian cancer remain well below the goal of universal testing (10, 11). Study showed that only one third of patients diagnosed with ovarian cancer had germline genetic testing (12). Many factors limit germline genetic testing in those with clinical indications, including the cost of germline genetic testing, limited access to genetic counseling and testing, and fear of familial discrimination (13, 14). In China, data on the real-world uptake of germline genetic testing among epithelial ovarian cancer patients remain limited. According to clinical practice, testing rates may fall short of guideline expectations, potentially due to barriers such as low patient awareness and socioeconomic disparities. Moreover, the key determinants influencing Chinese epithelial ovarian cancer patients’ decisions to undergo germline genetic testing have not been systematically elucidated.

A clear understanding of the germline genetic testing uptake and its determinants can inform targeted interventions to overcome barriers, align clinical practice with international guidelines, and ultimately optimize outcomes for both ovarian cancer patients and their at-risk family members. Against this backdrop, our study aimed to explore the awareness and uptake rate of germline genetic testing among Chinese women with epithelial ovarian cancer, and to analyze the determinants influencing their decision to undergo this testing.

## Methods

### Study subjects

A cross sectional study was conducted to investigate patients with epithelial ovarian cancer who visited the Obstetrics and Gynecology Hospital of Fudan University from November 2023 to September 2024. The inclusion criteria for the study subjects were: pathologically diagnosed with epithelial ovarian cancer; diagnosed for more than 1 month; older than 18 years of age; and provided informed consent to participate in the study. Patients were excluded if cancer histology was non-epithelial or exhibited impairments in language communication or cognitive function.

### Data collection

Data collection for this study was performed in the gynecologic oncology ward and daycare chemotherapy center. Ethical approval for the study was obtained from the Obstetrics and Gynecology Hospital of Fudan University Research Ethics Committee (approval number: 2023-101). All patients with epithelial ovarian cancer provided informed consent after being fully informed of the study purpose, procedures, and privacy protection measures.

Disease-related data were extracted from electronic medical records. The recorded variables included: operative time, histology, clinical stage (according to the International Federation of Gynecology and Obstetrics (FIGO) staging classification). A questionnaire was used to collect demographic information (education level, marital status, employment status, place of residence, average monthly income per capita, number of children, family history of cancer, etc.), patients’ awareness of germline genetic testing, current status of germline genetic testing, and reasons for not undergoing testing. The questionnaire was developed systematically based on previous published literature, professional guidelines, and expert consultation with gynecologic oncologists and genetic counseling specialists. A preliminary clinical survey involving 10 patients was conducted to revise ambiguous items, optimize wording, and ensure logical clarity.

The section assessing patients’ awareness of germline genetic testing comprised 11 items. Each scored on a 5-point Likert scale: “very well informed”(4 points), “fairly well informed” (3 points), “moderately informed” (2 points), “slightly informed” (1 points), and “not at all informed” (0 points). The total score ranged from 0 to 44. Consistent with widely adopted median-split stratification methods in similar health awareness studies, the total score was dichotomized using the median value as the cut-off point: scores ≥ 22 were defined as high awareness, and scores < 22 were defined as low awareness. After obtaining participants’ informed consent, questionnaires were distributed and collected on-site, with researchers providing timely responses to any questions raised during completion. Questionnaires with incomplete responses were excluded, and only valid fully completed questionnaires were enrolled in the final analysis.

### Statistical analysis

Statistical analyses were performed using SPSS 21.0 (IBM Corporation, Armonk, NY). Categorical data were presented as frequency and percentage, continuous data as mean and standard deviation (SD). Chi-square (χ²) test was used for univariate analysis, and binary logistic regression analysis was conducted for multivariate analysis. A p value of < 0.05 was considered statistically significant.

## Results

### Demographic and clinicopathological characteristics of patients with epithelial ovarian cancer

A total of 170 questionnaires were distributed in this study. Among these, 12 patients declined to complete the questionnaire, and 4 questionnaires were deemed invalid due to incorrect completion. Ultimately, 154 valid questionnaires were collected, with an effective recovery rate of 90.6%.

154 epithelial ovarian cancer patients were included in the analysis. Patients’ demographic characteristics are shown in **Table 1**. Most patients with ovarian cancer were diagnosed at advanced stages (stages III and IV) and had serous histology. Mean age at diagnosis was 55.92 ± 11.54 (range 21-80) years. Regarding family history of cancer, 66.23% of patients reported no family history of cancer, while 33.77% indicated having a family history of cancer. Specifically, 13.64% of the total patients had a family history of *BRCA*-related cancers including ovarian, breast, pancreatic, or prostate cancer, and 5.84% had a family history of colorectal or endometrial cancer.

**Table 1:** Baseline characteristics of patients with epithelial ovarian cancer.

Characteristic	n	%
Age (years)		
≤50	37	24.03
51~60	68	44.16
>60	49	31.82
Educational level		
High school and below	111	72.08
Bachelor’s degree and above	43	27.92
Marital status		
Unmarried	4	2.60
Married	142	92.21
Divorced	1	0.65
Widowed	7	4.55
Employment status		
Employed	24	15.58
Unemployed	33	21.43
Retired	97	62.99
Residence		
Rural	48	31.17
Urban	106	68.83
Monthly per capita household income (CNY)		
≤6000	87	56.49
>6000	67	43.51
Number of children		
0	8	5.19
1	110	71.43
2	32	20.78
≥3	4	2.60
Family history of cancer		
No	102	66.23
Yes	52	33.77
Family history of ovarian cancer, breast cancer, pancreatic cancer or prostate cancer		
No	133	86.36
Yes	21	13.64
Family history of colorectal cancer or endometrial cancer		
No	145	94.16
Yes	9	5.84
Histology		
Serous carcinoma	122	79.22
Mucinous carcinoma	7	4.55
Endometrioid carcinoma	8	5.19
Clear-cell carcinoma	17	11.04
FIGO Stage		
I	31	20.13
II	21	13.64
III	77	50.00
IV	25	16.23
Awareness of genetic testing		
Low awareness	97	62.99
High awareness	57	37.01

### Awareness of germline genetic testing among epithelial ovarian cancer patients

In terms of awareness regarding germline genetic testing among epithelial ovarian cancer patients, 62.99% demonstrated low awareness, while 37.01% exhibited a high level of awareness. The detailed distribution of their awareness levels is presented in **Table 2**. Regarding 11 key aspects of genetic testing, patients demonstrated relatively robust awareness of the link between genetic factors and ovarian cancer pathogenesis: the combined proportion of participants who were “very well informed” or “fairly well informed” was 39.61%, while only 6.49% reported being “not at all informed” about this association. Detailed knowledge about specific susceptibility genes and the appropriate candidates for genetic testing remained limited, only about 21% and 27% of respondents, respectively, reported being “very” or “fairly” informed in these two domains. Additionally, roughly 28% had no knowledge at all about how genetic counseling and testing can assist hereditary ovarian cancer families in risk management. Besides this, participates showed incomplete awareness of genetic testing’s clinical utility. For “genetic testing’s ability to identify pathogenic factors and estimate disease risk”, 9.09% were “very well informed,” 37.01% were “slightly informed,” and 14.29% were “not at all informed.” Regarding “genetic testing’s value in guiding treatment selection and predicting outcomes”, the corresponding proportions were 11.69%, 27.27%, and 14.94%. Awareness of hereditary risk to offspring was relatively higher, with about 71% possessing at least moderate understanding. In contrast, knowledge of preventive interventions was poorest: nearly 40% were unaware that risk-reducing surgery can prevent ovarian cancer in carriers, and almost 60% had no idea that preimplantation genetic testing can block transmission of susceptibility genes to the next generation.

**Table 2:** Awareness of genetic testing in patients with epithelial ovarian cancer n (%).

Contents	Very well-informed	Fairly well-informed	Moderately informed	Slightly informed	Not at all informed
1) Are you aware that the development of ovarian cancer is related to genetic factors?	24 (15.58)	37 (24.03)	42 (27.27)	41 (26.62)	10 (6.49)
2) Do you know which genes are associated with hereditary ovarian cancer?	6 (3.90)	26 (16.88)	32 (20.78)	60 (38.96)	30 (19.48)
3) Are you familiar with information about hereditary ovarian cancer genetic testing?	15 (9.74)	34 (22.08)	40 (25.97)	51 (33.12)	14 (9.09)
4) Do you know which individuals should undergo genetic counseling and testing?	13 (8.44)	29 (18.83)	31 (20.13)	58 (37.66)	23 (14.94)
5) Are you aware that genetic testing can identify ovarian cancer pathogenic factors and estimate disease risk?	14 (9.09)	29 (18.83)	32 (20.78)	57 (37.01)	22 (14.29)
6) Do you know that genetic testing can guide therapy selection and predict treatment outcomes?	18 (11.69)	43 (27.92)	28 (18.18)	42 (27.27)	23 (14.94)
7) Are you aware that genetic counseling and testing can help hereditary ovarian cancer families manage risk and lower cancer incidence?	7 (4.55)	22 (14.29)	18 (11.69)	64 (41.56)	43 (27.92)
8) Are you aware that germline genetic testing can identify hereditary ovarian cancer?	20 (12.99)	45 (29.22)	27 (17.53)	42 (27.27)	20 (12.99)
9) Are you aware that children of ovarian-cancer susceptibility-gene carriers are at risk of inheriting the mutation?	28 (18.18)	47 (30.52)	34 (22.08)	28 (18.18)	17 (11.04)
10) Are you aware that carriers of ovarian-cancer susceptibility genes can undergo risk-reducing surgery to prevent ovarian cancer?	8 (5.19)	19 (12.34)	22 (14.29)	44 (28.57)	61 (39.61)
11) Are you aware that preimplantation genetic testing can block transmission of susceptibility genes to the next generation?	4 (2.60)	7 (4.55)	11 (7.14)	40 (25.97)	92 (59.74)

### Uptake rate and influencing factors of germline genetic testing in epithelial ovarian cancer patients

In this study, 100 epithelial ovarian cancer patients underwent germline genetic testing, with an uptake rate of 64.94% among 154 eligible cases. Univariate analysis of factors influencing germline genetic testing uptake in epithelial ovarian cancer patients revealed statistically significant differences in the following variables: age, educational level, employment status, place of residence, average monthly household income, family history of *BRCA*-related cancers, and awareness of genetic testing (all p < 0.05). Details are presented in **Table 3**. Binary logistic regression analysis was conducted to identify factors associated with germline genetic testing uptake among patients, where “whether a patient underwent germline genetic testing” served as the dependent variable, and the statistically significant factors from the prior univariate analysis were included as independent variables. Results of multivariate logistic regression are shown in **Table 4**. Higher monthly per-capita household income was associated with a significantly increased likelihood of testing (OR = 3.30, 95 % CI 1.24-8.75, P = 0.017).Women with a family history of *BRCA*-related cancers were more likely to undergo testing (OR = 6.30, 95% CI 1.16-34.12, P = 0.033). Patients with high awareness of genetic testing were far more likely to be tested (OR = 8.81, 95 % CI 2.58-30.12, P = 0.001). Age, educational level, employment status, and residence were not significantly associated with germline genetic testing uptake (all P > 0.05).

**Table 3:** Univariate analysis of factors influencing germline genetic testing uptake in epithelial ovarian cancer patients.

Characteristic	Genetic testing (n=100)	Non-genetic testing (n=54)	χ^2^ value	*P* value
Age (years)			7.600	0.022
≤50	31 (31.00)	6 (11.11)		
51~60	40 (40.00)	28 (51.85)		
>60	29 (29.00)	20 (37.04)		
Educational level			11.677	0.001
High school and below	63 (63.00)	48 (88.89)		
Bachelor’s degree and above	37 (37.00)	6 (11.11)		
Marital status			2.236	0.525
Unmarried	3 (3.00)	1 (1.85)		
Married	93 (93.00)	49 (90.74)		
Divorced	1 (1.00)	0 (0.00)		
Widowed	3 (3.00)	4 (7.41)		
Employment status			12.069	0.002
Employed	22 (22.00)	2 (3.70)		
Unemployed	24 (24.00)	9 (16.67)		
Retired	54 (54.00)	43 (79.63)		
Residence			8.870	0.003
Rural	23 (23.00)	25 (46.30)		
Urban	77 (77.00)	29 (53.70)		
Monthly per capita household income (CNY)			15.328	0.000
≤6000	45 (45.00)	42 (77.78)		
>6000	55 (55.00)	12 (22.22)		
Number of children			2.621	0.454
0	7 (7.00)	1 (1.85)		
1	69 (69.00)	41 (75.93)		
2	22 (22.00)	10 (18.52)		
≥3	2 (2.00)	2 (3.70)		
Family history of cancer			3.493	0.062
No	61 (61.00)	41 (75.93)		
Yes	39 (39.00)	13 (24.07)		
Family history of ovarian cancer, breast cancer, pancreatic cancer or prostate cancer			6.967	0.008
No	81 (81.00)	52 (96.30)		
Yes	19 (19.00)	2 (3.70)		
Family history of colorectal cancer or endometrial cancer			0.000	1.000
No	94 (94.00)	51 (94.44)		
Yes	6 (6.00)	3 (5.56)		
Histology			2.515	0.473
Serous carcinoma	79 (79.00)	43 (79.63)		
Mucinous carcinoma	3 (3.00)	4 (7.41)		
Endometrioid carcinoma	5 (5.00)	3 (5.56)		
Clear-cell carcinoma	13 (13.00)	4 (7.41)		
FIGO stage			4.822	0.185
I	16 (16.00)	15 (27.78)		
II	13 (13.00)	8 (14.81)		
III	56 (56.00)	21 (38.89)		
IV	15 (15.00)	10 (18.52)		
Awareness of genetic testing			27.476	0.000
Low awareness	48 (48.00)	49 (90.74)		
High awareness	52 (52.00)	5 (9.26)		

**Table 4:** Multivariate logistic regression of determinants influencing germline genetic testing uptake in epithelial ovarian cancer patients (n=154).

Variable	B	S.E.	Wals	P value	OR	95% confidence interval
**Age(years)**(ref:≤50)			4.341	0.114		
51~60	-0.469	0.770	0.372	0.542	0.625	(0.138, 2.827)
>60	0.532	0.821	0.420	0.517	1.703	(0.341, 8.510)
**Educational Level**(ref: High school and below) Bachelor’s degree and above	0.506	0.650	0.605	0.437	1.658	(0.464, 5.927)
**Employment status** (ref: Employed)			2.515	0.284		
Unemployed	-0.963	1.023	0.887	0.346	0.382	(0.051, 2.833)
Retired	-1.471	0.962	2.341	0.126	0.230	(0.035, 1.512)
**Residence** (ref: Rural) Urban	-0.256	0.510	0.252	0.616	0.774	(0.285, 2.103)
**Monthly per capita household income(CNY)** (ref: ≤6000) >6000	1.193	0.498	5.733	0.017	3.296	(1.242, 8.751)
**Family history of ovarian, breast, pancreatic or prostate cancer** (ref: No) Yes	1.841	0.862	4.562	0.033	6.301	(1.164, 34.117)
**Awareness of genetic testing** (ref: Low awareness) High awareness	2.176	0.627	12.030	0.001	8.808	(2.576, 30.116)
**Constant**	0.763	1.079	0.500	0.479	2.145	

Bold values indicate variables with P<0.05 in univariate analysis, which were entered into the multivariate logistic regression model. ref = reference group.

### Pathogenic germline mutation status and carriers’ preventive and family screening intentions

Among the 100 epithelial ovarian cancer patients who underwent germline genetic testing, 3 had not yet received their test reports. Of the remaining 97 patients with available results, 19 (19.59%) were identified as carrying pathogenic or likely pathogenic germline mutations, comprising 15 cases of *BRCA* germline mutations, 2 *RAD51D* pathogenic mutations, and 1 each in *MSH6* and *MLH1* mutations. Among these 19 patients with detected germline mutations, 16 (84.2%) reported intent to undergo more frequent medical surveillance for secondary cancer prevention, while 3 (15.8%) indicated they would use preventive medications or surgery to mitigate risk of other related cancers. The majority of patients (18/19, 94.7%) shared their test results with family members, 16 patients (84.2%) would recommend their relatives to pursue cascade genetic testing, and 11 patients (57.9%) would advise their relatives to consider risk-reducing surgery if germline genetic testing confirms them as carriers.

### Reasons for refusal of germline genetic testing among epithelial ovarian cancer patients

54 epithelial ovarian cancer patients did not undergo germline genetic testing. Of these, 27 patients (50.0%) reported being unaware or unfamiliar with ovarian cancer germline genetic testing as the reason. 24 patients (44.4%) believed that germline genetic testing would not be beneficial for them, 16 patients (29.6%) indicated that germline genetic testing was too expensive to afford, and 3 patients (5.6%) expressed concerns about being unable to accept test results or worrying that results might affect their marriage and daily life.

## Discussion

### Summary of main results

Our findings highlight that epithelial ovarian cancer patients’ awareness of germline genetic testing is generally low, with significant imbalance across domains. Awareness was relatively better regarding foundational genetic associations with ovarian cancer and offspring hereditary risk, but limited for specific susceptibility genes and eligible candidates for testing. Awareness regarding the clinical utility of genetic testing and its role in risk management among families affected by hereditary ovarian cancer remains insufficient, with the lowest awareness for preventive interventions such as risk-reducing surgery for ovarian cancer prevention and preimplantation genetic testing to prevent the transmission of susceptibility genes to offspring.

We found that nearly two thirds of epithelial ovarian cancer patients had germline genetic testing. Higher monthly per-capita household income, a family history of *BRCA*-related cancers and higher awareness of germline genetic testing were associated with a significantly increased likelihood of testing.

19.6% of patients with epithelial ovarian cancer carried a pathogenic variant that warrants a potential adjustment in care, such as secondary breast cancer screening, risk-reducing surgery, cascade testing, or lifestyle interventions.

### Results in the context of published literature

Given that multiple clinical guidelines endorse germline testing and the popularization about genetic testing, the utilization rate of germline testing among patients with ovarian cancer has increased in recent years. However, our study showed that more than one-third of patients still do not receive testing promptly after diagnosis, we are still far from achieving the goal outlined in the guidelines, which is that “all patients with epithelial ovarian cancer should undergo germline genetic testing for *BRCA1*, *BRCA2*, and other ovarian cancer susceptibility genes.”

The germline genetic testing rate in our study places in the middle range when compared with international reports. Based on data from patients diagnosed with ovarian cancer in California and Georgia (USA) between 2013 and 2014, approximately one-third of patients (30.9%) underwent germline genetic testing (12). A 2022 real-world database from the United States reported that 59.5% of patients diagnosed with ovarian cancer had undergone germline *BRCA* testing (15). A collaborative evaluation of hereditary cancer genetics services across three gynecologic oncology clinics (USA, Brazil, Mexico) showed that 68.7%, 32.6%, and 60.2% of their ovarian cancer patients had completed germline genetic testing (16). A single-center Norway study from 2024 showed that, in the years 2008-2019, 56.2% patients with ovarian cancer were tested for *gBRCA* mutations, in the years 2014-2019, 72.3% received testing (17).Yet our rate remains lower than those observed in European parallel-testing initiatives and physician-led mainstream pathways. The UK’s SIGNPOST program, which integrates parallel germline-and-somatic testing within a multidisciplinary cancer clinical team, achieved the highest uptake at 97 % (1). The gynecologic oncologist-initiated genetic testing model in Canada McGill University Health Centre boosted testing from a historical 50.9 % to 86.2 % after implementation (18).

Our study found that per-capita monthly income influences the uptake of germline genetic testing. This indicates that lower income may impede patients from accessing testing services, covering testing costs, or receiving related genetic counseling, thereby affecting testing rates. This result is consistent with existing literatures, which demonstrated high-income families had a more positive attitude towards the genetic test than low-income families (19).Studies also reported a significant association between patients’ socioeconomic status and testing uptake: individuals with high socioeconomic status were more likely to complete the test compared to patients with low socioeconomic status (20, 21).

A family history of ovarian, breast, pancreatic or prostate cancer increased the odds of testing by more than sixfold (OR = 6.30, P = 0.033). Personal or familial experience drives hereditary cancer awareness, which in turn strongly motivates pursuit of genetic evaluation. This effect is biologically plausible: individuals with a family history of related cancers are more likely to perceive personal benefits from testing and are also more likely to be referred by clinicians for genetic counseling and testing. Other studies also demonstrated that ovarian cancer patients with a family history of breast or ovarian cancer were more likely to undergo genetic counseling and testing (22, 23). The first Japanese nationwide multicenter study of *BRCA* mutation testing in ovarian cancer also showed that patients with family history of ovarian cancer had a slightly higher prevalence of *gBRCA* mutations (24).

Patients’ awareness of genetic testing is a decisive factor in their decision-making. This finding is echoed in studies of mainstream genetic-testing pathways, where delivering testing information directly at the point of care substantially boosts patients’ acceptance of testing (18, 25, 26). In this study, we analyzed the reasons why epithelial ovarian cancer patients did not undergo genetic testing and found that the main causes were insufficient awareness or cognitive misunderstandings, including being unaware of or unfamiliar with ovarian cancer genetic testing, and believing that genetic testing would not be beneficial to themselves. Previous studies have demonstrated that ovarian cancer patients need a range of information to address the uncertainties and challenges that they encounter while taking genetic tests (27). Consequently, improving patients’ knowledge about genetic testing is a pivotal strategy for narrowing the testing gap.

From a translational oncology and precision medicine perspective, germline mutation detection yields direct and actionable clinical value, particularly for PARP inhibitor eligibility stratification and individualized therapeutic optimization. Germline *BRCA1/2* pathogenic variants represent the most validated predictive biomarkers for PARP inhibitor targeted therapy in EOC, as endorsed by global clinical guidelines and landmark clinical trials (28–31). Standardized germline testing enables accurate identification of eligible candidates for first-line maintenance therapy, recurrent disease salvage treatment, and postoperative adjuvant targeted intervention, thereby minimizing empirical medication and maximizing personalized therapeutic benefits.

This study also provides evidence to inform standardized familial cascade testing strategies for hereditary ovarian cancer. In our study, the majority of EOC patients with pathogenic germline mutations were willing to inform their family members of genetic test results and recommend at-risk relatives to undergo familial cascade genetic screening, demonstrating a positive behavioral basis for the clinical promotion of family-based genetic prevention programs. Notably, existing evidence indicates that nearly one-third of at-risk relatives remain uninformed of their risk of carrying a cancer-associated pathogenic variant (32), and only one-third of at-risk relatives within hereditary breast and ovarian cancer families ultimately complete cascade genetic testing (33). These results highlight a stark mismatch between patients’ positive intention for family screening and suboptimal real-world cascade testing uptake, thereby representing a critical missed opportunity for precision cancer prevention.

### Strengths and weaknesses

Our study offers several strengths. First, we integrated analysis of patient awareness, testing uptake, influencing factors, mutation outcomes, and reasons for non-testing, providing a holistic understanding of the current status of germline genetic testing in epithelial ovarian cancer patients, which is more systematic than most existing studies focusing solely on a single domain. Second, we combined univariate and multivariate logistic regression to control for potential confounding factors, enabling accurate identification of independent predictors of testing uptake. This design enhances the reliability and clinical relevance of the findings. Third, the detailed investigation of reasons for non-testing and behavioral intentions of mutation carriers directly addresses real-world barriers to testing, laying a practical foundation for developing targeted interventions. Despite these strengths, the study has inherent limitations. First, it was a single-center study carried out at an obstetrics and gynecology hospital in Shanghai, the findings may not fully reflect the overall situation nationwide. All awareness data were collected via patient self-report, inevitably leading to potential recall bias or reporting bias. Second, the sample size (n = 154) may limit statistical power, especially when detecting subtle effects of demographic variables such as age, education level, and occupational status, which increases the likelihood of false-negative findings. Moreover, the cross−sectional design precludes causal inference, for instance, awareness of genetic testing could act as both a driver of testing uptake and a consequence of having been tested.

### Implications for practice and future research

Our findings indicate that while the overall rate of germline genetic testing among epithelial ovarian cancer patients has improved, yet a substantial gap remains—over one-third of patients still do not undergo timely testing following diagnosis. This shortfall is influenced by socioeconomic factors, family history of relevant cancers, and limited patient awareness of genetic testing. These results underscore the need for targeted strategies to address testing barriers.

First, germline genetic testing should be fully integrated into routine diagnostic and therapeutic workflows for newly diagnosed EOC patients to establish a standardized clinical pathway. Clinicians should proactively initiate genetic screening at the early disease stage to identify pathogenic mutations in *BRCA* and other susceptibility genes, synchronously complete PARP inhibitor eligibility assessment, and ensure timely delivery of targeted maintenance or salvage therapy for mutation-positive patients to maximize survival benefits. Second, genetic counseling services should be strengthened for patients and at-risk families. Genetic counselors are recommended to provide result interpretation for mutation carriers, elaborate the clinical value of familial cascade testing, clarify the indications of risk-reducing surgery and preimplantation genetic testing, and correct prevalent cognitive biases toward germline genetic testing. Meanwhile, systematic professional training should be regularly conducted for healthcare providers to improve awareness, initiative, and standardization in initiating germline genetic testing, reducing missed screening cases caused by insufficient professional cognition. In addition, patient education programs and public health science campaigns should be promoted to popularize knowledge regarding hereditary ovarian cancer, the clinical significance of germline testing, the advantages of precision targeted therapy, and standardized familial prevention strategies, so as to comprehensively elevate public and patient awareness of genetic testing. Given the significant socioeconomic disparities in testing accessibility and the prominent financial burden for low-income patients, targeted health policy optimization is urgently required. We recommend expanding national medical insurance reimbursement coverage to include routine germline genetic testing for EOC patients, covering core susceptibility gene profiling to reduce individual out-of-pocket costs and achieve equitable access to precision genetic services.

For future research, nationwide multi-center cohort studies are needed to further validate our findings and improve the generalizability of the results. Such prospective studies are warranted to track changes in ovarian cancer patients’ genetic testing behaviors, the implementation of guideline-recommended preventive measures, and long-term clinical outcomes. This study design would also facilitate robust causal inference and assessment of the long-term clinical benefits of germline genetic testing in this patient population. Future research should also prioritize optimizing interventions to further enhance germline genetic testing uptake in ovarian cancer. Randomized controlled trials are needed to evaluate targeted approaches—such as mainstream testing models or evidence-based educational initiatives—and to identify cost-effective strategies that boost uptake while improving patient outcomes. Additionally, we recommend integrating timely psychological support into genetic testing pathways, as this will help address potential barriers to patient engagement.

## Conclusions

Among Chinese patients with epithelial ovarian cancer, awareness of germline genetic testing is generally low, with critical deficiencies in knowledge that directly inform clinical decision-making and familial risk management. Although nearly two-thirds of these patients had undergone germline genetic testing, a substantial gap persists between this uptake rate and the target outlined in clinical guidelines. Socioeconomic status, family history of associated cancers, and patients’ awareness of genetic testing were identified as the principal determinants of germline genetic testing utilization in this population. Targeted strategies are needed to address testing barriers, notably, enhancing patients’ awareness of genetic testing maybe a pivotal approach to narrowing the existing testing gap.

## Data Availability

The original contributions presented in the study are included in the article/supplementary material. Further inquiries can be directed to the corresponding author.
